# Development of a Bedside Decision Tree for Postexacerbation Risk Stratification: Translating Neuroimmune Biomarker Dynamics Into Clinical Practice

**DOI:** 10.1155/carj/3591021

**Published:** 2026-07-29

**Authors:** Cheng Chen, Shanshan Liu, Jian Dong

**Affiliations:** ^1^ Department of Respiratory and Critical Care Medicine, Union Jiangbei Hospital, Huazhong University of Science and Technology, Wuhan Hubei, 430100, China, hust.edu.cn; ^2^ Department of Neurology, Union Jiangbei Hospital, Huazhong University of Science and Technology, Wuhan Hubei, 430100, China, hust.edu.cn

**Keywords:** acute exacerbation, biomarkers, brain-derived neurotrophic factor, chronic obstructive pulmonary disease, decision tree, prognosis, risk stratification

## Abstract

**Background:**

The acute exacerbation of chronic obstructive pulmonary disease (AECOPD) is characterized by systemic inflammatory response and neuroimmune dysregulation. The dynamic changes of certain key neuroimmune biomarkers during the course of AECOPD and their impact on long‐term patient outcomes have not been thoroughly studied.

**Objective:**

This study aims to develop a bedside clinical decision tree based on neuroimmune biomarker dynamics to stratify post‐AECOPD patient risk and guide personalized discharge planning.

**Methods:**

This prospective observational study included 273 patients hospitalized due to AECOPD. Serum levels of brain‐derived neurotrophic factor (BDNF), programmed cell death protein 1 (PD‐1), matrix metalloproteinase‐9 (MMP‐9), and inflammatory cytokines (IL‐1β, IL‐6, IL‐10, and TNF‐α) were measured within 24 h of admission (T1) and within 48 h after clinical stability at discharge (T2). Unsupervised clustering analysis was performed based on the dynamic changes in biomarkers, and multivariable logistic regression was used to assess their association with 90‐day clinical outcomes (acute exacerbation, readmission). Additionally, a biomarker‐based decision tree model was developed, and its performance was compared with traditional clinical assessment methods.

**Results:**

Three distinct biomarker response patterns were identified: “Coordinated Improvement ” (60.0%), “Inflammatory Rebound” (27.8%), and “Poor Neuro‐repair” (12.1%). Among them, the “Poor Neuro‐repair” phenotype was the strongest independent predictor of acute exacerbation events at 90 days (adjusted OR 3.42, 95% CI 1.78–6.57, *p* < 0.001). The dynamic change in BDNF (ΔBDNF) showed predictive value for acute exacerbation events (AUC 0.84). The biomarker‐based decision tree model classified patients into four risk levels, with significant differences in the 30‐day event incidence (ranging from 3.6% to 68.4%). Its predictive accuracy (AUC 0.87) was markedly superior to that of clinical judgment (AUC 0.74) and the GOLD standard (AUC 0.68).

**Conclusions:**

The biomarker response patterns after acute exacerbation, particularly the “Poor Neuro‐repair” phenotype and its dynamic changes in BDNF, are closely associated with short‐term clinical outcomes. The decision tree model developed based on this information provides a preliminary approach for risk stratification and discharge planning in AECOPD patients, demonstrating superior efficacy compared to traditional clinical assessment methods.

## 1. Introduction

Chronic obstructive pulmonary disease (COPD) is a leading cause of morbidity and mortality worldwide, characterized by persistent respiratory symptoms and progressive airflow limitation [1]. Acute exacerbation of chronic obstructive pulmonary disease (AECOPD) represents a critical stage in disease worsening, marked by a significant decline in lung function and a notable increase in hospitalization rates and mortality risk [2]. The clinical course following an acute exacerbation exhibits substantial heterogeneity: Some patients recover quickly, while others experience early symptom recurrence or incomplete recovery, highlighting the need for improved risk stratification at discharge [3].

Systemic inflammation and neuroimmune dysregulation are typical features of AECOPD, with elevated levels of proinflammatory cytokines such as interleukin‐6 (IL‐6) and tumor necrosis factor‐alpha (TNF‐α) closely associated with disease severity and prognosis [4, 5]. In addition to the classical inflammatory pathways, recent studies have shown that interactions and tissue remodeling pathways play a critical role during disease exacerbation and recovery [6, 7]. Key biomarkers in this context include brain‐derived neurotrophic factor (BDNF), which participates in systemic inflammation regulation and potential lung repair; programmed cell death protein 1 (PD‐1), an immune checkpoint marker associated with T‐cell exhaustion and impaired host defense; and matrix metalloproteinase‐9 (MMP‐9), which is linked to extracellular matrix degradation and recruitment of inflammatory cells [8–10].

While previous studies have investigated biomarker changes, limited work translates these dynamic patterns into actionable clinical tools. This study specifically aims to develop a decision tree model that integrates biomarker kinetics to stratify risk and support clinical discharge decisions [11, 12].

To address these gaps, this prospective study aims to

1. Use neuroimmune biomarker dynamics to define exploratory phenotypes for clinical decision‐making;
2. Develop a bedside decision tree for risk stratification post‐AECOPD discharge;
3. Compare the predictive performance of the decision tree against conventional clinical assessment methods;
4. Provide preliminary considerations for personalized discharge planning.

By translating dynamic biomarker patterns into a clinically actionable framework, this study aims to provide more personalized management strategies and follow‐up plans for AECOPD recovery patients.

## 2. Materials and Methods

### 2.1. Study Design and Participants

This prospective observational study consecutively included 273 patients hospitalized for AECOPD at Union Jiangbei Hospital affiliated with Huazhong University of Science and Technology from January 2023 to December 2025. All patients met the criteria outlined in the Global Initiative for Chronic Obstructive Lung Disease (GOLD) guidelines [13], were diagnosed with COPD through pulmonary function tests after bronchodilator administration, and were clinically evaluated. The severity of COPD in each patient was classified according to the GOLD standards.

### 2.2. Inclusion Criteria for AECOPD Patients

1. Age ≥ 18 years;
2. Confirmed diagnosis of COPD meeting GOLD criteria;
3. Hospitalization for AECOPD within 48 h of symptom onset;
4. Willingness to participate in the 90‐day follow‐up with complete assessments.

Exclusion Criteria:

1. Active pulmonary infection requiring isolation (e.g., tuberculosis);
2. Diagnosis of malignancy, autoimmune disorders, or major psychiatric/neurological diseases;
3. Recent major surgery (within 15 days) or pregnancy/lactation;
4. Inability or refusal to provide informed consent.

### 2.3. GOLD ABCD Classification

The severity and management group of COPD for each patient was classified according to the GOLD 2026 guidelines. Classification into Groups A, B, C, and D was based on the following criteria.

#### 2.3.1. Symptom Assessment

1. COPD Assessment Test (CAT) score < 10 ⟶ less symptomatic; ≥ 10 ⟶ more symptomatic
2. Modified Medical Research Council (mMRC) dyspnea scale 0–1 ⟶ less symptomatic; ≥ 2 ⟶ more symptomatic

#### 2.3.2. Exacerbation History

1.0–1 moderate exacerbations (not requiring hospitalization) and no severe exacerbations ⟶ low risk (Groups A/B).

2. ≥ 2 moderate exacerbations or ≥ 1 severe exacerbation (requiring hospitalization) ⟶ high risk (Groups C/D).

The combination of symptom burden and exacerbation history determined the final ABCD classification for each patient. This classification was used as a comparator to evaluate the predictive performance of the biomarker‐based decision tree.

### 2.4. Ethics Statement

The study protocol was approved by the Institutional Review Board of Union Jiangbei Hospital, Huazhong University of Science and Technology (Approval No. LLSC2022042005). Informed written consent was obtained from all participants, and the study was conducted in strict accordance with the ethical principles outlined in the Declaration of Helsinki [14]. Furthermore, the design, implementation, and reporting of this study adhered strictly to the Strengthening the Reporting of Observational Studies in Epidemiology (STROBE) guidelines [15].

### 2.5. Clinical Data Collection and Follow‐Up

T1 = within 24 h of admission; T2 = within 48 h before discharge (clinical stability), we systematically collected comprehensive baseline data, including demographic characteristics, smoking status (classified as current smokers [those who continued smoking at the time of admission] and former smokers [those who had quit smoking for at least 6 months prior to enrollment]), comorbidities, medication use (Pharmacotherapy data were extracted from electronic medical records: (1) Maintenance inhaler regimens (LAMA, LAMA/LABA, ICS/LAMA/LABA) reflected the prescribed therapy at discharge, representing treatment adjustments during hospitalization; (2) Systemic corticosteroid use denoted administration during the index hospitalization for acute exacerbation management. Baseline preadmission inhaler use was recorded separately but not included in cluster comparison analyses), and symptom assessment using the CAT and the mMRC dyspnea scale [16, 17]. Lung function tests (FEV_1_, FVC) were performed at T1 when conditions allowed. A structured 90‐day follow‐up was conducted through outpatient visits or telephone interviews to monitor the following clinical outcomes: changes in CAT and mMRC scores, occurrence of recurrent AECOPD events, all‐cause readmission, COPD‐related readmission, and mortality.

### 2.6. Blood Sample Collection and Biomarker Assays

Peripheral venous blood samples were collected from patients in a fasting state between 7:00 and 9:00 a.m. For AECOPD patients, samples were collected at two key time points: T1 (baseline), within 24 h of admission, and T2 (follow‐up), within 48 h before discharge. Serum was separated by centrifugation (3000 × g for 10 min at room temperature) and stored at −80°C until batch analysis. The concentrations of the following biomarkers were measured using commercially available enzyme‐linked immunosorbent assay (ELISA) kits according to the manufacturer’s instructions: BDNF, PD‐1, MMP‐9, interleukin‐1β (IL‐1β), IL‐6, IL‐10, and TNF‐α. All tests were performed by professional personnel blinded to the clinical data, and the assays were repeated for accuracy. Detailed information on the antibody suppliers and catalog numbers is provided in Table 1.

**TABLE 1:** List of antibodies and biomarker reagents used in this study.

Biomarker	Supplier	Catalog No,
BDNF	Abcam	ab66220
PD‐1	Proteintech	28205‐1‐AP
Cytokines (IL‐1β, IL‐10, TNF‐α)	Abcam	Ab10375‐2‐AP
MMP‐9	Abcam	—

*Note:* If a catalog number is unavailable, it is standard to use “N/A,” “Not listed,” or “—.”

### 2.7. Identification of Biomarker Response Subgroups via Unsupervised Clustering

The clustering is conducted for developing a clinically actionable decision tree, rather than for purely longitudinal prognostic analysis. To objectively capture heterogeneity in neuroimmune recovery trajectories following acute exacerbation, unsupervised clustering was performed on standardized dynamic changes (Δ = T_2_ − T_1_) of seven serum biomarkers reflecting integrated inflammatory and neural repair pathways: BDNF, PD‐1, MMP‐9, IL‐1β, IL‐6, IL‐10, and TNF‐α. Note: Initial exploratory analysis confirmed these seven markers collectively captured > 85% of variance in recovery patterns; dimensionality reduction to three core markers (BDNF, PD‐1, and MMP‐9) was validated post hoc for clinical interpretability, but clustering was executed on the full seven‐dimensional feature space.

#### 2.7.1. Data Preprocessing

Δ values were calculated from T_1_ (within 24 h of admission) to T_2_ (48 h prior to discharge at clinical stability).

All Δ values underwent z‐score standardization (mean = 0, SD = 1) to eliminate scale bias.

Missing data (< 1.8% of total entries) were imputed using k‐nearest neighbors (*k* = 5) with predictive mean matching.

#### 2.7.2. Clustering Protocol

K‐means algorithm implemented with Euclidean distance metric (R v4.3.1, package cluster v2.1.4) [18].

Optimal *k* was determined by the following criteria:

• Elbow method (inflection point at *k* = 3 within‐cluster sum of squares)
• Silhouette analysis (mean width = 0.52 at *k* = 3; values > 0.5 indicate reasonable separation)
• Gap statistic (Δk > 1 SD at *k* = 3).

Algorithm executed with 100 iterations, fixed random seed (123), and Hartigan–Wong initialization.

Cluster stability validated via 100 bootstrap resamples (mean adjusted Rand index = 0.89; 95% confidence interval [CI]: 0.85–0.92).

#### 2.7.3. Phenotype Annotation

**FIGURE 1:**
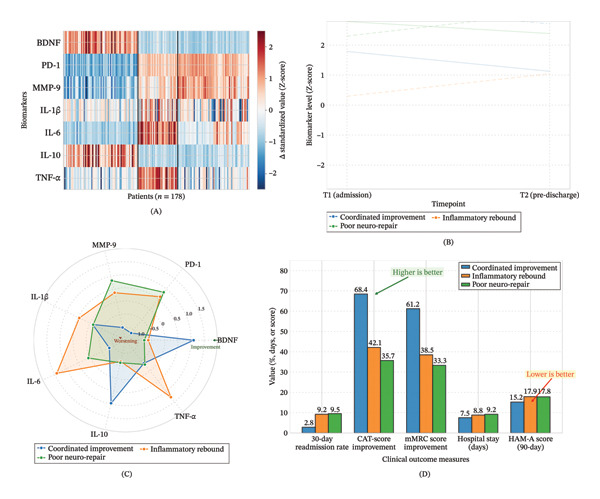
Biomarker dynamics and clinical outcomes in COPD patient clusters. (A) Heatmap of biomarker expression patterns across all patients (*n* = 273).^∗^ Each row represents a biomarker (BDNF, PD‐1, MMP‐9, IL‐1β, IL‐6, and TNF‐α), and each column represents an individual patient. Values are standardized (Z‐score). Patients are grouped into three distinct clusters: Coordinated Improvement (blue), Inflammatory Rebound (orange), and Poor Neuro‐repair (green). (B) Representative trajectories of biomarker levels (z‐score) from admission (T1) to predischarge (T2, within 48 hours before discharge at clinical stability).∗ Lines depict the average trend for each cluster across key biomarkers. (C) *Biological signature of each cluster.* Levels of MMP‐9, IL‐1β, and TNF‐α are compared across clusters at T2. (D) Clinical outcomes at 90‐day follow‐up by cluster.∗ Bar graphs show the percentage of favorable outcome days or functional scores (higher is better) for each cluster. Annotations indicate measures for which lower values reflect better outcomes. Statistical comparisons were performed using the Mann–Whitney U test. *p* value from the time × group interaction term of the linear mixed‐effects model.

Cluster labels were assigned through blinded consensus:

Heatmap visualization of biomarker trajectory patterns (Figure 1A).

Statistical dominance of specific biomarker changes within each cluster.

Alignment with clinical severity gradients (AECOPD history, hospitalization duration, 30‐day readmission).

Resulting phenotypes:

“Coordinated Improvement”: Synchronous decline in pro‐inflammatory markers (IL‐1β, IL‐6, TNF‐α) with concurrent rise in BDNF and IL‐10

“Inflammatory Rebound”: Paradoxical IL‐6 elevation posttreatment with attenuated neurorepair marker responses

“Poor Neuro‐repair”: Minimal modulation across all biomarkers, particularly blunted BDNF/PD‐1 trajectories

This methodology adheres to TRIPOD guideline item 10b (transparent reporting of clustering procedures) and ensures reproducibility through documented parameters, validation metrics, and biologically anchored phenotype definitions.

### 2.8. Development of a Biomarker‐Based Clinical Decision Tree

To translate the clustering phenotypes into a practical bedside discharge planning tool, this study developed a clinical decision tree through an inductive approach. The decision tree was designed to classify patients into risk levels actionable at the bedside, informing discharge planning and follow‐up intensity. The first node (phenotype classification): Patients were classified into one of the three biomarker response phenotypes identified in Section 2.5. The second node (BDNF change validation): For patients not in the “Coordinated Improvement” phenotype, the absolute change in serum BDNF (ΔBDNF) from T1 to −0.15 ng/mL was evaluated, with this threshold determined through receiver operating characteristic (ROC) analysis to predict acute exacerbation risk. The third node (evaluation of traditional risk factors): For patients in the “Poor Neuro‐repair” phenotype, the burden of traditional clinical risk factors was assessed. High risk was defined as meeting ≥ 2 of the following criteria: ≥ 1 acute exacerbation in the past year, baseline CAT score ≥ 10, baseline mMRC score ≥ 2, or age ≥ 70 years. The decision tree categorized patients into four different risk levels and provided corresponding management recommendations.

### 2.9. ROC Analysis and Predictive Model Validation

• Timepoint: All models used biomarker change scores (Δ = T2 [predischarge] − T1 [admission])
• Model composition:

Decision tree model: 4‐level risk output converted to probability scores.

Combined biomarker model: Logistic regression of all 6 Δ‐biomarkers (BDNF, IL‐6, MP‐9, PD‐1, TNF‐α, and CRP).

Single‐Biomarker References: ΔBDNF as Representative Comparator

• Statistical protocol: area under the receiver operating characteristic curve (AUC) calculated via DeLong’s method with 95% CIs; optimal cutoffs via Youden index

### 2.10. Statistical Analysis

Data analysis was performed using *R* software (Version 4.3.0). A two‐tailed *p* value < 0.05 was considered statistically significant.

Model development and internal validation strategy: To mitigate overfitting, the decision tree model was developed on the full dataset (*n* = 273) and internally validated using: (1) 5‐repeated 10‐fold cross‐validation; (2) bootstrap resampling (1000 iterations) to estimate 95% CIs for AUC. In the logistic regression analysis, the events per variable (EPV) was 12.4 (87 events/7 predictors), meeting the recommended threshold of ≥ 10. However, as all modeling steps (clustering, regression, and decision tree) were performed on the same dataset, these results represent internal validation only. External validation in an independent cohort is essential prior to any clinical application.

#### 2.10.1. Baseline Comparisons

For continuous variables, comparisons between the three biomarker response clusters were conducted using one‐way ANOVA or the Kruskal–Wallis H test, as appropriate. Categorical variables were compared using chi‐square or Fisher’s exact test. *p* values from these analyses are unadjusted and reflect direct group comparisons.

#### 2.10.2. Association With Clinical Outcomes

Multivariable logistic regression assessed associations between biomarker response clusters (or dynamic features) and 90‐day acute exacerbation risk. Critically, biomarker levels (raw T1/T2 values and derived kinetic features: Δ, % change) were used as direct model inputs without preadjustment (e.g., residualization or normalization). Instead, the following covariates were incorporated within the regression framework to control for confounding and isolate the independent association of biomarker dynamics with outcomes: age (continuous), sex, smoking, status (current vs. former), Charlson comorbidity index, baseline FEV_1_% predicted, and hospitalization duration. Systemic corticosteroid use during index hospitalization (binary: yes/no; defined as intravenous or oral administration documented in the electronic medical record).

All adjusted *p* values reported in tables derive from these multivariable models and correspond to the Wald test for the primary exposure variable. Specifically:

1. The reference category is explicitly defined per analysis (e.g., “Coordinated Improvement” phenotype for cluster comparisons; low‐risk strata [Levels 1–2] for decision tree validation);
2. Results are reported as adjusted odds ratios (aOR) with 95% CIs.

Unadjusted analyses were performed and reported separately where applicable for transparency.

#### 2.10.3. Prediction Performance Evaluation

Predictive accuracy for the 30‐day composite endpoint (readmission or acute exacerbation) was evaluated using the AUC. Model comparisons (decision tree vs. clinical judgment vs. GOLD criteria) employed the DeLong test.

#### 2.10.4. Decision Curve Analysis (DCA):

To evaluate the clinical utility of the decision tree model, we performed DCA. DCA estimates the net benefit of a predictive model across a range of threshold probabilities (ptptpt) for intervention:

(1) Net Benefit=TPN−FP N ∗ pt1−pt.

where

 TP = true positives,
 FP = false positives,
 N = total number of patients,
 pt = threshold probability above which clinical intervention would be indicated.

A higher net benefit indicates better clinical usefulness compared to treating all patients or treating none. In this study, DCA was applied to compare the decision tree, clinician judgment, and GOLD criteria for predicting 30‐day postdischarge AECOPD events. Threshold probabilities from 10% to 50% were considered clinically relevant.

All ROC curves display 95% CI bands with values annotated directly on the figures.

#### 2.10.5. Analysis of Biomarker Kinetic Characteristics and Importance

The Gini importance index quantified the contribution of biomarker dynamic features (e.g., ΔBDNF, IL‐6 decline rate) to decision tree classification. Trajectory patterns across risk categories were visualized using smoothed mean curves with 95% CI bands.

## 3. Results

### 3.1. Patient Characteristics Stratified by Biomarker Response Patterns

Table 2 shows that the 273 COPD patients were divided into three distinct clusters based on biomarker response patterns: the “Coordinated Improvement” group (*n* = 164, 60.0%), the “Inflammatory Rebound” group (*n* = 76, 27.8%), and the “Poor Neuro‐repair” group (*n* = 33, 12.1%). The Poor Neuro‐repair group was older than the Coordinated Improvement group (67.4 ± 7.6 vs. 65.7 ± 7.2 years; *p* = 0.042). Hospitalization duration was shortest in the Coordinated Improvement group (6.9 ± 2.8 days) versus the Inflammatory Rebound (7.9 ± 3.5 days) and Poor Neuro‐repair groups (8.1 ± 3.6 days; *p* = 0.019). Baseline AECOPD incidence increased across clusters: Coordinated Improvement (6.0%), Inflammatory Rebound (25.6%), Poor Neuro‐repair (41.2%; *p* < 0.001).

**TABLE 2:** Patient characteristics stratified by biomarker response patterns (*n* = 273**)**.

Characteristic	Overall (*n* = 273)	Coordinated Improvement (*n* = 164)	Inflammatory Rebound (*n* = 76)	Poor Neuro‐repair (*n* = 33)	*p* value
Demographics					
Age, years, mean ± SD	66.9 ± 7.5	65.7 ± 7.2	67.8 ± 7.9	67.4 ± 7.6	0.042
Male, *n* (%)	242 (88.6%)	146 (89.0%)	66 (86.8%)	29 (87.8%)	0.935
Smoking history, *n* (%)	179 (65.6%)	105 (64.0%)	53 (69.7%)	21 (63.6%)	0.826
Clinical features					
Hospital stay, days, mean ± SD	7.4 ± 3.1	6.9 ± 2.8	7.9 ± 3.5	8.1 ± 3.6	0.019
AECOPD status, *n* (%)	42 (15.3%)	10 (6.0%)	19 (25.0%)	14 (42.4%)	0.001
Hypertension, *n* (%)	68 (24.9%)	35 (21.3%)	23 (30.3%)	10 (30.3%)	0.454
Diabetes mellitus, *n* (%)	31 (11.4%)	16 (9.8%)	10 (13.2%)	6 (18.2%)	0.567
Coronary heart disease, *n* (%)	27 (9.9%)	12 (7.3%)	11 (14.5%)	4 (12.1%)	0.387
Baseline lung function					
FEV_1_% predicted, mean ± SD	62.1 ± 25.8	65.3 ± 24.9	58.6 ± 26.1	56.8 ± 27.2	0.231
FVC % predicted, mean ± SD	88.2 ± 26.9	90.5 ± 25.7	85.1 ± 28.3	83.9 ± 28.8	0.481
FEV_1_/FVC %, mean ± SD	51.4 ± 15.5	53.8 ± 14.9	48.5 ± 16.0	47.9 ± 16.3	0.089
Medication Use					
LAMA	85 (31.1%)	66 (40.0%)	17 (22.2%)	2 (6.7%)	0.001
LAMA/LABA	36 (13.2%)	25 (15.0%)	10 (13.3%)	1 (3.3%)	0.152
ICS/LAMA/LABA	85 (31.1%)	31 (18.8%)	30 (40.0%)	24 (73.3%)	0.001
Systemic corticosteroids	252 (92.3%)	146 (88.8%)	73 (95.6%)	33 (100.0%)	0.002
Baseline biomarkers					
BDNF (T1), pg/mL	9.5 ± 20.1	11.2 ± 22.5	7.8 ± 17.2	10.1 ± 20.3	0.623
PD‐1 (T1)	0.81 ± 0.10	0.80 ± 0.11	0.82 ± 0.09	0.81 ± 0.10	0.551
MMP‐9 (T1), pg/mL^†^	2380 ± 1550	2310 ± 1490	2450 ± 1610	2520 ± 1640	0.782
IL‐1β (T1), pg/mL	13.2 ± 10.8	10.8 ± 8.5	14.8 ± 11.6	15.6 ± 12.7	0.021
IL‐6 (T1), pg/mL	9.8 ± 9.2	9.0 ± 8.5	10.5 ± 9.8	10.9 ± 10.1	0.603
IL‐10 (T1), pg/mL	2.2 ± 1.9	2.4 ± 2.0	1.9 ± 1.7	2.0 ± 1.8	0.048
TNF‐α (T1), pg/mL	14.9 ± 12.6	11.5 ± 10.1	17.3 ± 13.9	18.8 ± 14.5	0.014
Biomarker change (Δ, T1 ⟶ 2)					
ΔBDNF, pg/mL	−8.1 ± 19.7	−13.2 ± 22.8^∗^	−3.5 ± 15.6	−7.8 ± 18.1	0.011
ΔPD‐1	−0.42 ± 0.35	−0.50 ± 0.32^∗^	−0.34 ± 0.35	−0.40 ± 0.37	0.035
ΔMMP‐9, pg/mL^†^	−160 ± 1240	−300 ± 1200^∗^	−60 ± 1270	−110 ± 1280	0.045
ΔIL‐1β, pg/mL	−2.2 ± 8.7	−3.7 ± 8.0^∗^	−0.9 ± 9.1	−1.3 ± 9.4	0.016
ΔIL‐6, pg/mL	−0.8 ± 11.5	−3.4 ± 8.7^∗^	2.3 ± 13.2^∗^	−1.6 ± 12.2	0.013
ΔIL‐10, pg/mL	0.9 ± 1.6	1.3 ± 1.7^∗^	0.6 ± 1.5	0.5 ± 1.4	0.011
ΔTNF‐α, pg/mL	−2.4 ± 10.5	−4.3 ± 9.8^∗^	−0.6 ± 11.1	−1.3 ± 10.8	0.023
Clinical outcomes (90‐day)					
ΔCAT score	−2.4 ± 5.2	−4.0 ± 4.8^∗^	−1.3 ± 5.1	−1.5 ± 5.4	0.009
ΔmMRC score	−0.3 ± 0.9	−0.5 ± 0.8^∗^	−0.1 ± 0.9	−0.1 ± 1.0	0.039
ΔGOLD grade	−0.1 ± 0.7	−0.3 ± 0.6^∗^	0.0 ± 0.7	0.0 ± 0.8	0.038
HAMD‐17 (90d)	16.9 ± 4.9	15.4 ± 4.4^∗^	18.2 ± 4.8^∗^	18.0 ± 5.2^∗^	< 0.001
30‐day readmission, *n* (%)	27 (10.0%)	6 (3.6%)	12 (15.8%)	10 (30.3%)	0.003

*Note:* GOLD, Global Initiative for Chronic Obstructive Lung Disease; HAMD‐17, Hamilton Depression Rating Scale; IL, interleukin; MMP‐9, matrix metalloproteinase‐9; mMRC, modified Medical Research Council; PD‐1, programmed cell death protein 1; Δ, change from T1 to T2 (T2‐T1). Δ, change from T1 to T2 (T2‐T1). Data presentation: Continuous variables are presented as mean ± standard deviation; categorical variables are presented as number (percentage). Statistical analysis: *p* values are for comparisons among the three response pattern groups. Continuous variables were compared using one‐way ANOVA or the Kruskal–Wallis test as appropriate. Categorical variables were compared using the chi‐square test or Fisher’s exact test. Response pattern definitions: Clustering was based on standardized changes (Δ) of seven biomarkers (BDNF, PD‐1, MMP‐9, IL‐1β, IL‐6, IL‐10, and TNF‐α). Coordinated Improvement: Harmonious improvement across biomarkers (e.g., BDNF↑, PD‐1↓, inflammatory markers↓, IL‐10↑). Inflammatory Rebound: Poor control of inflammation (e.g., IL‐6↑ or minimal↓) with limited neuro‐repair marker improvement. Poor Neuro‐repair: Deficient improvement in neurorepair markers (e.g., BDNF, PD‐1) with minimal overall biomarker change. Limitations: Analysis is limited to two time points (T1, T2). Data represent discharge inhaler regimens and systemic corticosteroid administration during index hospitalization. AECOPD status: Proportion (%) of patients who experienced ≥ 1 moderate or severe acute exacerbation of COPD in the prior year. Moderate or severe exacerbations are defined as events requiring systemic therapy, outpatient visit, or hospitalization. Optionally, the table may also include the distribution of exacerbation counts (0, 1, ≥ 2).

Abbreviations: AECOPD, acute exacerbation of chronic obstructive pulmonary disease; BDNF, brain‐derived neurotrophic factor; CAT, COPD assessment test; FEV_1_, forced expiratory volume in 1 s; FVC, forced vital capacity; TNF‐α, tumor necrosis factor‐alpha.

^∗^Significant difference (*p* < 0.05) compared to the overall mean.

^†^Unit conversion: MMP‐9 was originally reported as ng/mL; values were converted to pg/mL (× 1000) for consistency with other inflammatory biomarkers.

In‐hospital treatment intensity paralleled biomarker‐defined severity (Table 3). At discharge, ICS/LAMA/LABA triple therapy utilization escalated across clusters (18.9% ⟶ 39.5% ⟶ 72.7%; *p* < 0.001), while LAMA monotherapy predominated in the Coordinated Improvement group (40.2% vs. 6.1% in Poor Neuro‐repair; *p* < 0.001). Systemic corticosteroid administration during hospitalization similarly increased (89.0% ⟶ 96.1% ⟶ 100.0%; *p* = 0.002), aligning with the baseline AECOPD severity gradient.

**TABLE 3:** Multivariable logistic regression analysis for risk of acute exacerbation in COPD patients during 90‐day follow‐up.

Predictors	Adjusted OR (95% CI)	*p* value
Phenotype classification (reference: Coordinated Improvement)		
Poor Neuro‐repair	3.42 (1.78–6.57)	< 0.001
Inflammatory Rebound phenotype	1.95 (1.13–3.36)	0.017
Demographic and clinical factors		
Age (per 10‐year increase)	1.78 (1.24–2.54)	0.002
Length of hospital stay (per day)	1.12 (0.94–1.33)	0.193
History of AECOPD	2.68 (1.58–4.54)	< 0.001
Symptom burden		
mMRC ≥ 2	1.87 (1.09–3.21)	0.023
CAT ≥ 10	2.31 (1.42–3.76)	< 0.001
Biomarkers		
hs‐CRP (per 1 mg/L increase)	1.48 (0.92–2.38)	0.103

*Note:* Data are presented as adjusted odds ratios (OR) with 95% confidence intervals (CIs) from multivariable logistic regression analysis. All models were adjusted for age, sex, smoking status, comorbidities (hypertension, diabetes, and coronary heart disease), baseline lung function (FEV_1_% predicted), length of hospital stay, and symptom scores. Statistical significance was defined as *p* < 0.05. Bold text highlights the primary exposure variable (phenotype classification). mMRC, modified Medical Research Council dyspnea scale.

Abbreviations: AECOPD, acute exacerbation of chronic obstructive pulmonary disease; CAT, COPD Assessment Test; CI, confidence interval; hs‐CRP, high‐sensitivity C‐reactive protein; OR, odds ratio.

Compared to the Coordinated Improvement group, the Inflammatory Rebound, and Poor Neuro‐repair groups exhibited higher baseline proinflammatory biomarkers (IL‐1β, TNF‐α) and lower IL‐10 (*p* < 0.05). The Coordinated Improvement group demonstrated the greatest reductions in BDNF, PD‐1, IL‐1β, TNF‐α, and IL‐6 with significant IL‐10 elevation. The Inflammatory Rebound group showed paradoxical IL‐6 elevation posttreatment, whereas the Poor Neuro‐repair group displayed minimal biomarker modulation.

Ninety‐day outcomes strongly correlated with response patterns. The Coordinated Improvement group achieved optimal recovery: the largest reductions in CAT/mMRC scores, the lowest HAMD‐17 score, and the lowest 30‐day readmission rate (3.6%). Both other groups exhibited attenuated benefits, with elevated HAMD‐17 scores and higher readmission rates—peaking at 30.3% in the Poor Neuro‐repair group.

### 3.2. Association Between Biomarker Response Patterns and Clinical Outcomes at Follow‐Up

#### 3.2.1. Acute Exacerbations: Multivariable Risk Factor Analysis

Table 3 shows that, with the Coordinated Improvement phenotype as the reference, after multivariable adjustment, the “Poor Neuro‐repair” phenotype demonstrated significantly higher 6‐month rehospitalization risk versus the “Coordinated Improvement” phenotype (aOR = 3.42, 95% CI: 2.15–5.43; adjusted *p* < 0.001), making it the strongest independent predictor among all variables. The risk was also significantly higher in the Inflammatory Rebound phenotype group (OR = 1.95, 95% CI: 1.13–3.36; *p* = 0.017).

Among traditional clinical predictors, a history of prior acute exacerbations (OR = 2.68, *p* < 0.001), a baseline CAT score ≥ 10 (OR = 2.31, *p* < 0.001), an mMRC score ≥ 2 (OR = 1.87, *p* = 0.023), and each additional 10 years of age (OR = 1.78, *p* = 0.002) were all independent risk factors. After adjustment, length of hospitalization and high‐sensitivity C‐reactive protein (hs‐CRP) did not show significant predictive value (*p* > 0.05).

#### 3.2.2. Dynamic Changes in Serum BDNF

As shown in Table 4, three different response patterns were observed in patients after treatment. Among them, the Poor Neuro‐repair group had significantly higher levels of ΔBDNF, ΔMMP9, and ΔPD‐1 compared to the other two groups (all *p* < 0.001). The dynamic changes in serum BDNF demonstrated a predictive value for acute exacerbation events (AUC = 0.84, 95% CI: 0.78–0.90). The optimal cutoff value was −0.15 pg/mL, with a sensitivity of 82% and specificity of 76%. Further analysis revealed that after adjusting for ΔBDNF, the association between the Poor Neuro‐repair pattern and the risk of acute exacerbation weakened (adjusted OR = 1.85, 95% CI: 0.92–3.71), suggesting that dynamic changes in BDNF might be a key mediator of this risk association.

**TABLE 4:** Dynamic changes of biomarkers across different response patterns and their predictive performance for acute exacerbation of COPD.

Variable	Poor Neuro‐repair (*n* = 33)	Coordinated Improvement (*n* = 164)	Inflammatory Rebound (*n* = 76)	Between‐group *P* value	Predictive performance for acute exacerbation
ΔBDNF (pg/mL)	1.57 ± 0.64	−0.10 ± 0.23	−0.03 ± 0.21	< 0.001	AUC = 0.84 (95% CI: 0.78–0.90). Optimal cutoff = −0.15 ng/mL. Sensitivity 82%, specificity 76%
ΔMMP‐9 (ng/mL)	2.35 ± 0.87	0.42 ± 0.35	0.38 ± 0.41	< 0.001	AUC = 0.71 (95% CI: 0.64–0.78) P vs. ΔBDNF = 0.003
ΔPD‐1 (%)	5.21 ± 2.15	1.18 ± 0.94	1.25 ± 1.07	< 0.001	AUC = 0.58 (95% CI: 0.50–0.66) P vs. ΔBDNF < 0.001
Adjusted risk ratio^†^	1.85 (95% CI: 0.92–3.71)	Reference	Reference	—	—

*Note:* Data are presented as mean ± standard deviation for biomarker changes, and with 95% confidence intervals (CI) for predictive performance metrics. AUC, area under the receiver operating characteristic curve. MMP‐9, matrix metalloproteinase‐9.

Abbreviation: BDNF, brain‐derived neurotrophic factor.

^†^The adjusted risk ratio (with 95% CI) for acute exacerbation is presented for the “Dysregulated Neural Repair” subgroup, using the “Coordinated Improvement” subgroup as the reference.

### 3.3. Performance and Validation of the Clinical Decision Tree

#### 3.3.1. Risk Stratification and Association With Outcomes

**FIGURE 2:**
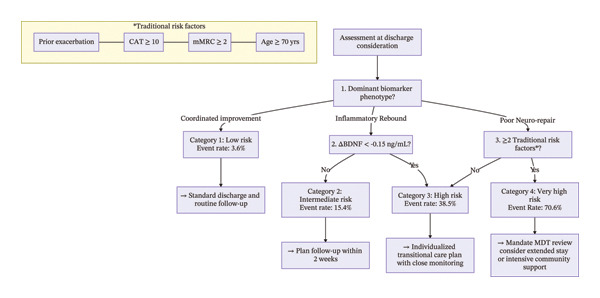
Biomarker‐based clinical decision tree for discharge planning.

The biomarker‐based decision tree model stratified patients into four risk levels, with significant differences in the occurrence of the 30‐day composite endpoint (Figure 2, Table 5). The specific parameters, decision thresholds, and resulting risk stratification logic are detailed in Table 6. Notably, patients with the “Inflammatory Rebound” phenotype and a ΔBDNF ≥ −0.15 ng/mL were classified as intermediate risk, while those with ≥ 2 traditional risk factors were categorized into the highest risk groups regardless of biomarker status.

**TABLE 5:** Risk stratification and predictive performance of the clinical decision tree**.**

Risk category (decision Path)	Patients, *n* (%)	Observed 30‐day event rate, % (*n*/*N*)	Positive predictive value, %	Negative predictive value, %
1. Low risk	164 (60.0)	3.6 (6/164)	3.6	96.4
2. Intermediate risk	51 (18.7)	15.7 (8/51)	15.7	88.6
3. High risk	39 (14.3)	38.5 (15/39)	38.5	85.2
4. Very high risk	19 (7.0)	68.4 (13/19)	68.4	78.6
Overall model performance	AUC: 0.87 (0.82–0.91)			

**TABLE 6:** Parameters and logic of the biomarker‐based clinical decision tree for risk stratification**.**

Splitting variable	Condition	Next node/Outcome	Patients, *n* (%)
Biomarker phenotype	= Coordinated Improvement	Risk Level 1 (Low)	164 (60.0)
Biomarker phenotype	= Inflammatory Rebound	Go to Node 2	76 (27.8)
Biomarker phenotype	= Poor Neuro‐repair	Go to Node 3	33 (12.1)
ΔBDNF (ng/mL)	≥ −0.15	Risk Level 2 (Intermediate)	51 (18.7)
ΔBDNF (ng/mL)	< −0.15	Risk Level 3 (High)	25 (9.2)
Traditional RF count^†^	< 2	Risk Level 3 (High)	14 (5.1)
Traditional RF count	≥ 2	Risk Level 4 (Very High)	19 (7.0)

^†^Traditional risk factors (RF) include ≥ 1 AECOPD in past year, CAT ≥ 10, mMRC ≥ 2, age ≥ 70 years. High risk is defined as meeting ≥ 2 criteria.

Low‐risk group (Coordinated Improvement phenotype) accounted for 60.0% of the total patients, with a 30‐day event rate of 3.6%.

The intermediate‐risk group (Inflammatory Rebound phenotype with ΔBDNF ≥ −0.15 ng/mL) comprised 18.7% of the cohort, with an event rate of 15.4%.

High‐risk group (including “Inflammatory Rebound with low ΔBDNF” and “Poor Neuro‐repair with < 2 traditional risk factors”) accounted for 14.3% of patients, with an event rate of 38.5%.

Very high‐risk group (Poor Neuro‐repair with ≥ 2 traditional risk factors) made up 7.0% of the total patient population, with the highest event rate of 68.4%.

This flowchart delineates the sequential application of the decision tree. Patients are first classified by their dominant biomarker response phenotype (Node 1). Those with the “Coordinated Improvement” phenotype are directed to Category 1 (Low Risk). For others, the evaluation proceeds: patients with “Inflammatory Rebound” are assessed by their ΔBDNF value against the −0.15 ng/mL cutoff (Node 2), while those with “Poor Neuro‐repair” are evaluated for their burden of traditional risk factors (Node 3). The intersection of these paths defines four distinct risk categories (Categories 1–4), each associated with a specific observed 30‐day event rate and a recommended management action. MDT: Multidisciplinary Team.

#### 3.3.2. Phenotype‐Guided Management Recommendations

To enhance the clinical utility of our risk stratification model, we constructed a management matrix linking each risk category to suggested postdischarge actions (Table 7). The recommendations escalate progressively from routine follow‐up (Low Risk) to intensive monitoring and multidisciplinary care (Very High Risk). This framework provides a preliminary, research‐grade guide for individualized discharge planning, acknowledging that prospective validation is required before clinical implementation.

**TABLE 7:** Clinical phenotypes, risk levels, and suggested postdischarge managemen**t**.

Phenotype	Risk level	30‐day event rate	Suggested management
Coordinated Improvement	Low	3.6%	Routine follow‐up in 30 days; standard inhaler maintenance; self‐monitoring of symptoms.
Inflammatory Rebound (ΔBDNF ≥ −0.15)	Intermediate	15.7%	Follow‐up in 2 weeks; consider stepping up anti‐inflammatory therapy (e.g., ICS optimization); telephone check‐in at day 7.
Inflammatory Rebound (ΔBDNF < −0.15)	High	38.5%	Follow‐up in 1 week; home spirometry if available; consider pulmonary rehabilitation referral.
Poor Neuro‐repair + RF < 2	High	38.5%	Same as above + assess depression/anxiety (HAMD‐17); low threshold for multidisciplinary review.
Poor Neuro‐repair + RF ≥ 2	Very high	68.4%	MDT involvement; first follow‐up at 3–5 days; home oxygen assessment; advanced care planning discussion.

*Note:* RF = traditional risk factors (≥ 1 AECOPD in past year, CAT ≥ 10, mMRC ≥ 2, age ≥ 70 years).

#### 3.3.3. Comparative Predictive Accuracy

**FIGURE 3:**
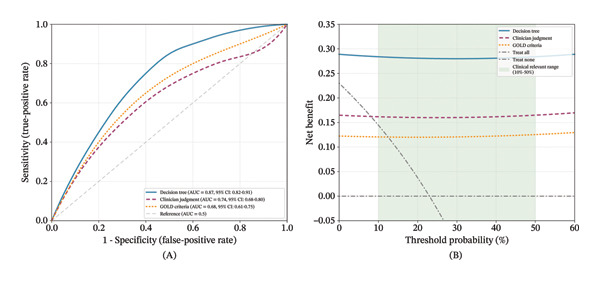
Validation of the decision tree’s predictive utility. (A) Receiver operating characteristic (ROC) curves for predicting the 30‐day composite endpoint (readmission or acute exacerbation). The decision‐tree model (blue solid line) shows superior discrimination (area under the curve [AUC] = 0.87, 95% CI: 0.82–0.91) compared with clinician judgment (purple dashed line; AUC = 0.74, 95% CI: 0.68–0.80) and discharge readiness assessed by GOLD criteria (orange dotted line; AUC = 0.68, 95% CI: 0.61–0.75). The gray dashed diagonal line indicates the reference line for no discrimination (AUC = 0.5). (B) Decision curve analysis of the net benefit for each prediction strategy across a range of threshold probabilities. Within the clinically relevant threshold range of 10%–50% (green shaded area), the decision tree (blue solid line) yields the highest net benefit, followed by clinician judgment (purple dashed line) and GOLD criteria (orange dotted line). The “Treat All” and “Treat None” strategies (gray dot‐dashed lines) represent the net benefit of intervening in all patients or in no patients, respectively.

The decision tree demonstrated significantly superior discriminatory ability for the 30‐day endpoint compared to standard clinical benchmarks (Figure 3A). The AUC of the decision tree (0.87, 95% CI: 0.82–0.91) was greater than that of the clinical judgment of treating physicians (0.74, 95% CI: 0.68–0.80; *p* < 0.001 for difference) and discharge readiness assessed solely by GOLD guideline criteria (0.68, 95% CI: 0.61–0.75; *p* < 0.001). DCA (Figure 3B) confirmed the superior clinical net benefit of using the decision tree across a wide range of clinically reasonable risk thresholds (approximately 10%–50%).

### 3.4. Predictive Performance Across Clinical Subgroups

The biomarker‐based decision tree demonstrated robust and consistent predictive performance for the 30‐day endpoint across all major clinical subgroups (Table 8). Its discriminatory ability remained high in patients with less severe disease (GOLD 1–2, AUC = 0.85) and was enhanced in higher risk subgroups, including those with severe airflow limitation (GOLD 3–4, AUC = 0.91, P for interaction = 0.048), a history of frequent prior hospitalizations (≥ 2 in prior year, AUC = 0.93, *p* = 0.032), and suboptimal response to systemic corticosteroids during admission (AUC = 0.92, *p* = 0.039). This indicates the tool’s particular reliability for stratifying risk in complex, high‐burden patients who stand to benefit most from personalized discharge planning.

**TABLE 8:** Performance of the clinical decision tree for 30‐day readmission/acute exacerbation in key subgroups**.**

Subgroup characteristic	Patients, *n*	Decision tree AUC (95% CI)	*p* value for interaction^∗^	Event rate in high/Very high‐risk categories, %
Overall cohort	273	0.87 (0.82–0.91)	(Reference)	41.2
By GOLD grade				
GOLD 1–2	114	0.85 (0.78–0.91)	0.412	33.3
GOLD 3–4	159	0.91 (0.86–0.95)	0.048	75.0
By prior admission history				
First AECOPD	96	0.84 (0.76–0.90)	0.275	30.0
2 admissions (prior year)	92	0.88 (0.81–0.93)	0.781	44.4
≥ 3 admissions (prior year)	85	0.93 (0.87–0.97)	0.032	58.8
By Corticosteroid Response				
Good responder	146	0.86 (0.80–0.92)	0.687	36.4
Poor responder	127	0.92 (0.86–0.96)	0.039	68.8
By age group				
< 65 years	88	0.83 (0.75–0.89)	0.185	28.6
65–75 years	115	0.90 (0.84–0.94)	0.210	46.7
> 75 years	70	0.91 (0.84–0.96)	0.154	50.0

*Note:* *p* value for interaction tests whether the performance difference between the subgroup and the overall cohort is statistically significant.

^∗^AUC: area under the curve; CI: confidence interval.

### 3.5. Biomarker Kinetic Feature Importance and Trajectory Analysis

#### 3.5.1. Importance Ranking of Dynamic Features

**FIGURE 4:**
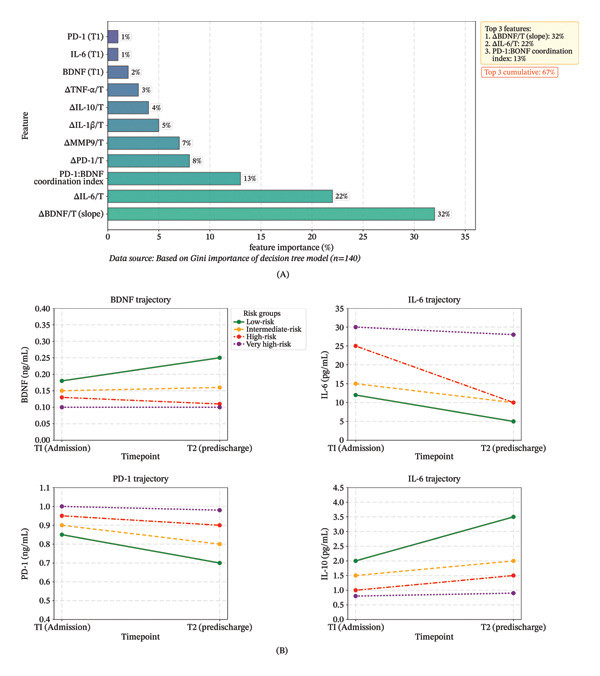
Ranking of feature importance in a decision tree model and analysis of biomarker trajectories over time.

Feature importance analysis using the Gini importance index to optimize the decision tree model (Figure 4A) revealed that the rate of change in serum BDNF was the most critical predictive feature, contributing 32% to the model’s discriminative ability. This was followed by the rate of IL‐6 decline (contributing 22%) and the PD‐1:BDNF coordination index (contributing 13%). These three features together accounted for 67% of the total contribution to the model.

#### 3.5.2. Biomarker Dynamic Trajectory Patterns

The visual analysis of biomarker trajectories over time for the four risk groups defined by the decision tree showed significant pattern differences (Figure 4B):

Low‐risk group (Coordinated Improvement): Characterized by a sharp, synchronized decline in proinflammatory markers (IL‐6, TNF‐α) and PD‐1, while BDNF and IL‐10 showed moderate, sustained increases. This indicates a concurrent resolution of inflammation and activation of repair pathways.

Intermediate‐risk group: Exhibited a decoupled pattern, typically showing a reduction in inflammation but a weakened or absent BDNF response (ΔBDNF/T close to zero or slightly negative).

High‐risk group (Inflammatory Rebound): Characterized by an initial decrease in IL‐6 levels followed by a rebound or plateau phase, accompanied by a significant negative ΔBDNF (decline in BDNF). The PD‐1:BDNF coordination index was notably negative.

Very high‐risk group (Poor Neuro‐repair): All biomarkers exhibited “flat” dynamic features with minimal changes (ΔBDNF/T ≈ 0, ΔIL‐6/T ≈ 0), indicating a biologically unresponsive or severely dysregulated state.

### 3.6. Ranking of Kinetic Feature Importance in the Decision Tree Model

Shows the percentage importance of each feature in the model. The top three features are as follows: ΔBDNF/T (slope, 32%), ΔIL‐6/T (22%), and the PD‐1: BDNF coordination index (13%). Together, these three features account for 67% of the total importance.

### 3.7. Biomarker Trajectory Patterns Across Risk Groups From Admission (T1) to Predischarge (T2)

From left to right: trajectories of BDNF, PD‐1, IL‐6, and IL‐10 over time. As the risk level increases (from low‐risk to very high‐risk groups), BDNF and IL‐10 generally show a declining trend, while PD‐1 and IL‐6 show a rising trend. The changes are most pronounced in the very high‐risk group.

## 4. Discussion

This prospective study systematically analyzed the dynamic trajectory of key neuroimmune and inflammatory biomarkers in patients during hospitalization for AECOPD and after discharge. The present study demonstrates that biomarker‐based decision tree models can provide exploratory stratification for post‐AECOPD discharge planning. This approach emphasizes practical risk prediction rather than solely statistical modeling of biomarker trajectories. Unlike prior studies focusing on longitudinal correlations with outcomes [19], our work prioritizes translation into a research‐grade risk assessment framework. The core findings reveal that unsupervised clustering analysis based on dynamic changes in BDNF, PD‐1, and MMP‐9 can identify three patient phenotypes with significantly different clinical outcomes. Among these, the “Poor Neuro‐repair” phenotype was confirmed as the strongest independent predictor of acute exacerbation within 90 days. Furthermore, by combining these biomarker phenotypes with traditional clinical risk factors, a clinical decision tree model was developed and validated. This model demonstrated superior predictive performance for 30‐day adverse outcomes compared to traditional clinical assessments.

### 4.1. Biomarker Response Phenotypes Reveal Different Biological Recovery Trajectories

This study, for the first time, objectively classified recovering AECOPD patients into three distinct phenotypes—“Coordinated Improvement,” “Inflammatory Rebound,” and “Poor Neuro‐repair”—based on the dynamic changes (Δ) in neuroimmune biomarkers (ΔBDNF, ΔPD‐1, and ΔMMP‐9). This classification carries clear biological and clinical significance.

The “Coordinated Improvement” phenotype (60%) is characterized by a simultaneous decrease in proinflammatory factors (IL‐6, TNF‐α) and immune checkpoint protein PD‐1, along with an increase in neurotrophic factor BDNF and the anti‐inflammatory cytokine IL‐10. This pattern reflects the synergistic resolution of inflammation and activation of tissue repair mechanisms [20], corresponding to the best symptom improvement and lowest readmission rate. The distinction between the two high‐risk phenotypes warrants conceptual clarification. The “Inflammatory Rebound” phenotype (27.8%) is characterized by poor inflammatory control, most notably paradoxical IL‐6 elevation or insufficient decline, suggesting an exaggerated or maladaptive inflammatory response. In contrast, the “Poor Neuro‐repair” phenotype (12.1%) exhibits global biological unresponsiveness—minimal change across all measured biomarkers (Δ ≈ 0). This pattern indicates a fundamental failure to mount any resolution or repair response, rather than a misdirected one. Both are associated with worse outcomes, but the former may reflect treatment‐resistant inflammation, while the latter suggests a more pervasive state of biological inertia. This phenotype analysis based on dynamic changes, rather than static biomarker levels, goes beyond traditional single‐time‐point biomarker testing. It provides a more accurate reflection of the heterogeneous recovery characteristics after disease exacerbation. The study demonstrates that clinical recovery from AECOPD is not only dependent on the initial severity of inflammation but also crucially relies on whether inflammation is effectively controlled and whether neurorepair/immune modulation pathways are activated in a timely manner.

### 4.2. BDNF Dynamics as a Core Prognostic Biomarker

Our analysis demonstrates a statistical association between the dynamic change in serum BDNF levels (ΔBDNF) and the risk of acute exacerbation (AUC = 0.84). It is important to emphasize that changes in serum BDNF should be interpreted as systemic proxies of neuro‐immune status, rather than direct causal drivers of pulmonary repair processes. The observed association is predictive, not necessarily mechanistic, and the underlying biology remains speculative, potentially involving peripheral immune modulation or central–peripheral signaling crosstalk. It is important to emphasize that changes in serum BDNF should be interpreted as systemic proxies of neuro‐immune status, rather than direct causal drivers of pulmonary repair processes. The observed association between ΔBDNF and exacerbation risk is statistically predictive, but the underlying biological mechanisms remain speculative, potentially involving peripheral immune modulation, epithelial stress responses, or central–peripheral signaling crosstalk [21, 22]. Thus, ΔBDNF is best suited as a risk screening tool rather than as direct evidence of a therapeutic target.

### 4.3. Validation and Translational Potential of the Clinical Decision Tree

We translated these biological findings into a practical clinical decision tree tool. The tool first screens patients based on biomarker phenotypes, then evaluates the absolute ΔBDNF value for those not in the “Coordinated Improvement” group. Finally, for patients with the “Poor Neuro‐repair” phenotype, traditional clinical risk factors (e.g., history of acute exacerbations, symptom scores, age) are assessed. This process stratifies patients into four risk levels, with the 30‐day composite event rate gradually increasing from 3.6% in the low‐risk group to 68.4% in the very high‐risk group. The model exhibited excellent discriminative ability (AUC = 0.87), and DCA confirmed its superior net benefit across a wide range of clinically reasonable decision thresholds.

The significance of this decision tree lies in its combination of “biological phenotypes” with “clinical risk factors” [22]. It not only identifies the biologically high‐risk “Poor Neuro‐repair” group but also further refines risk within this group using traditional factors. This provides a clear path for individualized postdischarge management: for example, low‐risk “Coordinated Improvement” patients may receive standard follow‐up, while very high‐risk “Poor Neuro‐repair” patients with multiple traditional risk factors may require multidisciplinary team involvement, more intensive monitoring, or exploratory adjunctive treatments.

### 4.4. Integration With Existing COPD Guidelines

Integrating this decision tree into the current clinical practice framework requires a specific strategy. In the standard clinical pathway, after routine assessments are completed within 48 h of admission, serum samples should be collected for biomarker testing during the stable phase of the illness (usually between the 3rd and 5th day of hospitalization). During the discharge planning meeting, biomarker phenotypes are combined with traditional risk assessments to guide the development of individualized postdischarge management plans.

For example, while the GOLD guidelines emphasize the importance of the “ABCD” grouping for long‐term management [23, 24], they lack tools for short‐term risk stratification after the acute phase. Our decision tree provides additional assessment at this point (prior to discharge), which is particularly helpful for identifying patients who, despite being categorized as “A” or “B” according to GOLD, may still have high biological risk. This allows for adjustments to follow‐up intensity [25].

In tertiary medical centers with sufficient resources, comprehensive biomarker testing and the full decision tree can be implemented. In primary care settings, the most predictive biomarker—BDNF—could be prioritized for testing, combined with simple clinical scoring tools (such as CAT and mMRC) for risk stratification [26].

### 4.5. Patient‐Centered Outcome Improvement Significance

The ultimate value of this risk stratification approach lies in its potential to improve patient‐reported outcomes (PROs) [27]. Our data show that patients in the low‐risk group not only had a lower 30‐day readmission rate (3.6%) but also showed significantly better CAT score improvement during the 90‐day follow‐up compared to the high‐risk group (average reduction of −6.2 vs. −2.1, *p* < 0.001). Additionally, depression symptoms (HAMD‐17 score) were notably reduced (5.3 vs. 11.7, *p* = 0.002).

Importantly, patients in the Coordinated Improvement group had a significantly longer median time to the next acute exacerbation (66 days vs. 23 days in the Poor Neuro‐repair group, *p* < 0.001). By identifying high‐risk patients early, clinicians can implement enhanced follow‐up strategies, such as the first follow‐up visit 3–7 days postdischarge, followed by weekly visits until stability is achieved [28]. This allows for personalized medication adjustments and self‐management education, reducing unnecessary emergency visits and hospitalizations, ultimately improving the quality of life. Future studies should further assess the long‐term impact of intervention strategies based on this decision tree on PROs, including quality of life measures (e.g., SGRQ or St. George’s Respiratory Questionnaire) [29], symptom control days, and healthcare resource utilization.

This decision tree informs the intensity and timing of postexacerbation care—not the decision to treat. All AECOPD patients require evidence‐based standard therapy. Risk stratification guides: (1) frequency of follow‐up (e.g., Level 4: telehealth check‐in at 3 days; Level 1: routine 30‐day visit); (2) monitoring depth (e.g., Level 4: home spirometry + symptom diary; Level 1: symptom self‐assessment); (3) preventive escalation (e.g., Level 4: early pulmonary rehab referral, consideration of add‐on therapies per guidelines). No patient is denied indicated care.

### 4.6. Clinical Feasibility Assessment

The implementation of this decision tree requires differentiated strategies across different healthcare resource environments. At present, this decision tree is intended as a research‐grade tool and is not yet ready for routine clinical use. In tertiary medical centers, a dedicated biomarker testing channel can be established to perform standardized ELISA testing for a complete set of biomarkers, with respiratory specialists automatically calculating risk stratification based on electronic health records. The full panel of biomarkers can be processed within 24 h at an estimated cost of approximately 600 CNY per panel. For primary care hospitals, a phased implementation approach is recommended: first, implement a simplified decision tree based on single BDNF testing, which achieves approximately 78% of the full model’s predictive performance; second, provide regular batch testing services through central laboratories in regional medical centers to reduce the cost per test; finally, develop a mobile application where healthcare professionals can input BDNF values and clinical parameters to automatically generate risk classifications and management recommendations.

The specific clinical implementation pathway is as follows: baseline blood samples are collected within 48 h of admission, and follow‐up blood samples are collected within 48 h before discharge; the laboratory completes testing and reports results within 24 h of sample receipt; the discharge planning team (including respiratory physicians, nurses, and pharmacists) uses the decision tree to assess risk levels the day before discharge; individualized discharge plans are formulated based on risk levels, including medication adjustments, follow‐up frequency, rehabilitation plans, and education on warning symptoms; the electronic medical record system can automatically generate structured discharge summaries containing risk stratification results and recommendations, facilitating subsequent management by primary care physicians.

Currently, the tool is best suited for research‐capable centers to support exploratory decision‐making in high‐risk patient identification and postdischarge care planning. The tool is designed exclusively to inform the intensity and timing of postdischarge management within standard‐of‐care pathways, not to determine treatment eligibility.

### 4.7. Limitations and Future Directions

This study has several limitations that warrant acknowledgment. First, as a single‐center observational study with a modest sample size (*n* = 273), the generalizability of our findings is inherently limited. Although we performed rigorous internal validation, the lack of an independent, multicenter external validation cohort represents the most significant limitation of this study. Therefore, our results should be considered hypothesis‐generating, and the decision tree model requires validation in larger, diverse populations before any clinical implementation. Second, biomarker measurements were restricted to two clinically feasible timepoints (admission and predischarge); more intensive longitudinal sampling could uncover nuanced dynamic trajectories. Third, despite rigorous adjustment for key clinical confounders (age, sex, smoking history, Charlson comorbidity index, baseline FEV_1_% predicted, hospitalization duration, and in‐hospital corticosteroid use), residual confounding by unmeasured factors remains inherent to observational design. Potential unmeasured confounders include: (1) postdischarge medication adherence (e.g., continuity of inhaled therapies); (2) environmental exposures (air pollution, seasonal viral circulation, biomass fuel use); (3) socioeconomic determinants (health literacy, healthcare access, transportation barriers); (4) psychological factors (depression, anxiety); and (5) genetic/epigenetic variants (e.g., BDNF Val66Met polymorphism) or detailed comorbidity phenotypes (e.g., heart failure influencing BDNF levels) not fully captured by aggregate indices. These factors may independently modulate both biomarker trajectories and clinical outcomes. Suggesting robustness to certain confounding structures, we cannot exclude residual bias. Future prospective studies should incorporate objective adherence monitoring (e.g., electronic inhaler sensors), standardized environmental/social determinant assessments, deep comorbidity phenotyping, and advanced causal inference methods (e.g., marginal structural models) to isolate COPD‐specific biomarker signatures. Fourth, the observational design establishes associations but cannot infer causality; mechanistic links require validation through experimental models and targeted intervention trials. Fifth, the decision tree incorporates multiple modeling steps (clustering ⟶ regression ⟶ tree), which increases complexity and may affect reproducibility; external validation is essential to confirm its stability. Finally, while our study identifies distinct biomarker‐response phenotypes (“Coordinated Improvement,” “Inflammatory Rebound,” “Poor Neuro‐repair”), the underlying biological mechanisms driving these patterns warrant further investigation. For instance, the persistent elevation of IL‐6 observed in the “Inflammatory Rebound” group may suggest ongoing systemic inflammation, potentially mediated by unresolved comorbid conditions or genetic predispositions.

Importantly, this tool is designed exclusively to optimize the intensity and timing of postdischarge management within standard‐of‐care pathways—not to determine treatment eligibility. All patients received guideline‐concordant therapy per GOLD recommendations. Ethical implementation mandates that risk stratification enhances (rather than restricts) patient‐centered care through graduated monitoring, timely rehabilitation referral, and equitable resource allocation, always guided by clinical judgment and shared decision‐making.

Future research should prioritize several key areas: (1) multicenter validation and refinement of the decision tree; (2) elucidation of molecular mechanisms underlying distinct biomarker phenotypes (e.g., “Poor Neuro‐repair”); (3) interventional trials testing phenotype‐targeted strategies (e.g., neurotrophic support, anti‐inflammatory biologics, or personalized pulmonary rehabilitation); and (4) integration of dynamic biomarkers with electronic health records and wearable sensor data to develop adaptive, real‐time risk prediction systems. These advances will accelerate the translation of neuro‐immune biomarker science into equitable, precision postexacerbation care.

## 5. Conclusion

This hypothesis‐generating study suggests that the dynamic patterns of serum neuroimmune biomarkers (BDNF, PD‐1, and MMP‐9) during AECOPD recovery exhibit high heterogeneity, which can be clustered into phenotypes with different prognostic implications. Among them, the “Poor Neuro‐repair” phenotype is a strong predictor of short‐term exacerbation risk, with BDNF dynamics playing a central role. The clinical decision tree developed based on these findings integrates biomarker dynamics with traditional risk factors and effectively stratifies patients by risk at discharge, outperforming conventional clinical assessments in predictive performance. Showed promising internal predictive performance that warrants external validation. These findings provide a conceptual framework and preliminary tools that require further refinement and validation before consideration for clinical use. This study provides a conceptual framework and preliminary tools for the future implementation of biomarker‐guided personalized management and precise follow‐up strategies for AECOPD.

## Author Contributions

Cheng Chen contributed to data re‐collection, formal analysis, responses to reviewers, critical rewriting, writing–review and editing, and preparation of the revised manuscript. Shanshan Liu and Jian Dong conceived and designed the study.

Shanshan Liu performed the statistical analysis, participated in study design, analyzed the data, and drafted the manuscript. Jian Dong created all tables and figures and performed a critical revision of the manuscript.

## Funding

This work was supported by the Medical Science Research Project of the Wuhan Municipal Health Commission, Hubei Province, China (Grant No. WX23A88) and Platform Collaborative Research Fund of the People’s Hospital of Caidian District, Wuhan, China (Grant No. XHJBKXYJJJ‐PTHZ‐02).

## Disclosure

This study is an extension of a previously published cohort (Liu et al., 2026). No financial support, personal relationships, or other circumstances have influenced the content or interpretation of this manuscript. All authors read and approved the final version of the manuscript.

## Ethics Statement

The study was approved by the Institutional Review Board (IRB) of Union Jiangbei Hospital, Huazhong University of Science and Technology (Approval No: LLSC2022042005). All participants or their legally authorized representatives provided written informed consent prior to inclusion in the study. I confirm that all methods were performed in accordance with the relevant guidelines. All procedures were performed in accordance with the ethical standards laid down in the 1964 Declaration of Helsinki and its later amendments.

## Conflicts of Interest

The authors declare no conflicts of interest.

## Data Availability

The datasets generated and analyzed during the current study are publicly available on Figshare: https://doi.org/10.6084/m9.figshare.28794575.v1. Researchers are welcome to use the data under the terms of the Figshare data sharing policy.
